# Acute piriformis syndrome in a military pilot with chronic lumbar radiculopathy: A case report

**DOI:** 10.1097/MD.0000000000045641

**Published:** 2025-11-14

**Authors:** Do Yun Kwon, Dong Hyuck Kim

**Affiliations:** a Department of Aviation Medical Squadron, Republic of Korea Air Force, Daegu, Republic of Korea; b Department of Anesthesiology, Daegu Catholic University Medical Center, Daegu, Republic of Korea; c Department of Anesthesiology and Pain Medicine, Daegu Catholic University School of Medicine, Daegu, Republic of Korea.

**Keywords:** neuropathic pain, piriformis syndrome, sciatic nerve compression, ultrasound-guided injection

## Abstract

**Rationale:**

Piriformis syndrome (PS) is a neuromuscular condition in which irritation or dysfunction of the piriformis muscle leads to compression of the sciatic nerve, resulting in radiating pain in the buttock and lower limb. Accurate diagnosis is often complicated by the clinical overlap between PS and other neurologic conditions such as lumbar radiculopathy or sciatica, as these disorders may present with similar symptomatology.

**Patient concerns:**

This case report describes the diagnosis and treatment of PS in a 37-year-old male military pilot. The patient had a history of lumbar disc herniation and experienced an acute tingling sensation in his left lower extremities following a high gravitational force (G-force) flight sortie.

**Diagnoses:**

PS was diagnosed based on ultrasonographic findings, which revealed a piriformis muscle thickness of 1.03 cm.

**Interventions:**

The patient initially underwent a blinded trigger point injection targeting the left piriformis muscle, which yielded partial pain relief. Nevertheless, due to ongoing radiating pain in the lower extremity, the patient was referred to a tertiary outpatient clinic, where an ultrasound-guided injection into the piriformis muscle was subsequently performed.

**Outcomes:**

An ultrasound-guided piriformis injection was administered, leading to a significant decrease in pain and enabling the patient to resume flight sorties.

**Lessons:**

This case highlights the importance of accurately diagnosing and treating PS in patients with lumbar disc herniation and suggests that ultrasound-guided piriformis injection can be an effective treatment method.

## 1. Introduction

Piriformis syndrome (PS) affects the piriformis muscle, a small muscle deep within the buttock that aids in external hip rotation and abduction.^[1]^ When overused or irritated, the piriformis muscle can compress or irritate the adjacent sciatic nerve, leading to pain in the buttocks, thighs, and legs.^[1,2]^ PS is diagnosed in 2% to 6% of patients with complaints of low back pain or sciatica; however, one study reported an incidence as high as 17.2%.^[2,3]^

Physical examination, in combination with clinical information, is used to diagnose PS, whereas imaging studies, such as magnetic resonance imaging or ultrasound, can be utilized to rule out other diseases.^[2]^ However, differentiating PS from other neurological pain-causing lesions, such as lumbar radiculopathy or sciatica, can be challenging because various diseases can mimic PS symptoms.^[2,4]^

Military pilots frequently report chronic back pain and radiating pain attributed to discomfort in body positioning and high gravitational force (G-force).^[5,6]^ Given the common occurrence of lumbar disc herniation in military pilots compared to the normal age group, physicians might not adequately consider the possibility of PS when diagnosing radiating lower-extremity pain in pilots, often suspecting lumbar disc herniation. Accordingly, we report a case of PS in a military fighter pilot patient with a history of chronic lumbar radiculopathy. This case report aimed to address the gap in the literature regarding the diagnosis of PS in military pilots. By presenting a detailed case study of a military fighter pilot with a history of chronic lumbar radiculopathy, we sought to contribute to the understanding of PS in this specific population. Our objective was to shed light on the diagnostic challenges faced by physicians and the potential impact of misdiagnoses, with implications for both pilot health and flight safety. This case illustrates the complexities involved in diagnosing PS in a high-performance occupational setting and emphasizes the need for tailored diagnostic approaches in such populations.

## 2. Case Report

A 37-year-old male military pilot, F-15, visited a military emergency room complaining of an acute tingling sensation in his left lower extremities. Two days prior, he had performed basic fighter maneuvers during dogfighting (a fight between 2 military aircrafts in which they fly very fast and very close to each other). While performing the mission, he applied excessive force to his lower extremities to overcome the high G force.

The patient had a history of lumbar nucleus pulposus (L-HNP) during training as an aviation cadet and had experienced neuropathic pain controlled with conservative treatment, including self-exercise and transforaminal epidural steroid injections, for 10 years. Symptoms were present during excessive physical activity, but did not interfere with daily living.

A month before the current visit, the patient had experienced low back pain and radiating pain in the right lower extremity, prompting an emergency room visit at the military base. Physical examination revealed no significant sensory changes in the right lower extremities. While the flexion, adduction, and internal rotation (FAIR) test results were negative, the straight leg raise (SLR) test results were positive for the right leg. The patient was transferred to a university hospital, where a C-arm-guided transforaminal epidural injection was administered. The patient experienced no pain during daily life or flight sorties with the use of pregabalin and non-steroidal anti-inflammatory drugs (NSAIDs).

Physical examination revealed no sensory impairment, and deep tendon reflexes of the knee and ankle were normal. Unlike a month ago, Lasègue’s sign, Freiberg sign, and Pace sign were positive in the left lower extremity, whereas the results of SLR and femoral nerve stretch tests were negative in both lower extremities. Direct tenderness of the left piriformis muscle is observed. Plain radiography of the hip and lumbar spine revealed no abnormalities. The resting and motion visual analog scale scores were 5 and 6 points, respectively. PS was suspected, leading to a blinded trigger point injection on the left piriformis muscle with 1 cc of 2% lidocaine and 4 cc of normal saline. Piriformis stretching exercises were prescribed, while medications for lumbar radiculopathy, including pregabalin and NSAIDs, were continued.

Two days after initiating treatment, there was some improvement in pain; however, the patient still experienced remnant lower extremity radiating pain and was subsequently referred to a tertiary referral outpatient clinic at the Department of Anesthesiology. Physical examination at the outpatient clinic showed negative Lasègue’s, Freiberg, and Pace signs; however, mild tenderness of the left piriformis muscle with slight discomfort during the FAIR test was observed.

**Figure 1.**
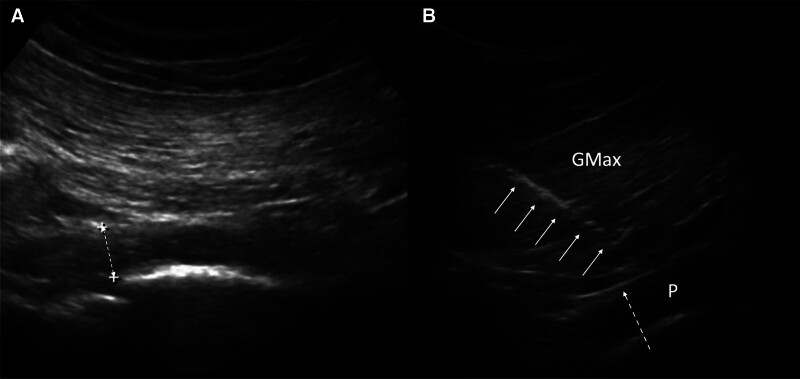
Muscle identification and injection. (A) The gluteus maximus and piriformis muscle were identified using a curved probe. The thickness of the piriformis muscle was measured using ultrasound (dotted 2-way arrow). (B) An ultrasound-guided injection was performed, targeting the piriformis muscle and fascia between the gluteus maximus and piriformis muscle. White arrows indicate the needle, and dotted arrows indicate fascia. GMax = gluteus maximus muscle, P = piriformis muscle.

After identifying the surface landmarks, including the posterior superior iliac spine (PSIS), sacral hiatus (SH), and greater trochanter (GT), the following anatomical guidance is applied: The line drawn between the PSIS and SH approximates the lateral border of the sacrum. The second line is defined by connecting the midpoint of the lateral sacral border to the upper border of the GT. A low-frequency curvilinear probe (2.5–5 MHz, Philips HD11 XE, Philps Medical Systems) was positioned along this line to visualize the piriformis muscle, which lies beneath the gluteus maximus and superficial to the sciatic nerve. A probe was used to confirm the location of the ischial spine. The piriformis muscle was confirmed through external and internal hip rotations with a measured thickness of 1.03 cm (Fig. 1A). No anatomical variations were observed in the sciatic or piriformis muscle. Ultrasound-guided injections were performed using 2.5 mL of 2% lidocaine and 7.5 mL of normal saline. Regimens were injected into the interfacial layer and the subfascial piriformis muscle using an in-plane technique on the long-axis view of the piriformis muscle (Fig. 1B).

One week after the ultrasound-guided injection, the resting and motion visual analog scale scores for lower extremity pain decreased to 2 points, and the patient had no difficulty in performing flight sorties. Two weeks after the injection, the patient experienced no discomfort, except for mild hyperesthesia in the left lateral foot (feeling of unusual hypersensitive touch when wearing socks). Three months later, the patient had no pain or sensory deficit and had no issues in performing routine tasks or flight sorties.

## 3. Discussion

This case report describes the treatment outcomes of a 37-year-old male pilot diagnosed with PS following a high-G-force flight sortie. The patient received ultrasound-guided piriformis injection, which proved to be an effective method for PS treatment.

The piriformis primarily functions as a hip abduction and external rotation muscle, contacting the roots of the first, second, and third sacral nerves.^[7]^ Therefore, abnormal conditions of the piriformis due to trauma or inflammation can cause irritation of the sciatic nerve, which can lead to lower extremity pain. One study demonstrated that the sciatic nerve can be directly compressed by spasm or inflammation of the piriformis muscle,^[8]^ whereas another study reported that only in some cases where the sciatic nerve passes through the piriformis can muscle fibers stimulate the nerve and cause neuropathy.^[9]^

The diagnosis of PS is rather challenging because there are no definitive physical examinations, tests, or imaging studies to confirm the diagnosis. Hopayian and Danielyan identified 4 key symptoms that define PS: History of trauma, pain at the sacroiliac joint, exacerbation with sitting, and tender or palpable mass in the buttock.^[10]^ The Lasègue sign (pain elicited by applying pressure to the piriformis muscle when the hip is flexed at 90° and the knee is extended), Freiberg sign (pain during passive internal rotation of the hip), Pace sign (pain and weakness during resisted abduction and external rotation of the hip in a flexed or sitting position), and FAIR test are commonly used to aid in the diagnosis of piriformis syndrome (PS).^[10,11]^ The patient in this case had a known history of L-HNP. During a visit 1 month prior, the physical examination revealed negative findings for the Lasègue sign, Freiberg sign, Pace sign, and FAIR test, while the SLR test was positive. Based on these findings, the pain was attributed to L-HNP. However, after exposure to high G-force, a subsequent physical examination showed positive findings for the Lasègue sign, Freiberg sign, Pace sign, and tenderness in the piriformis muscle, whereas the previously positive SLR test results were negative. These findings suggest that the current source of pain is more likely to be PS than L-HNP.

Electrodiagnostic studies can be used to differentiate PS from other neuropathies.^[11]^ Additionally, they may assist in diagnosing PS by evaluating the H-reflex in the FAIR position or by analyzing the wave amplitude and conduction delay of the peroneal H-reflex.^[12,13]^ However, there is currently insufficient evidence supporting the definitive diagnostic criteria for these methods, and electrodiagnostic studies are not routinely employed in the diagnosis of PS. Furthermore, given the patient’s history of a lumbar herniated nucleus pulposus (HNP), it is difficult to determine whether the denervation potentials observed on the electromyogram are attributable to the previous HNP or to a compressive neuropathy caused by PS.^[14]^ Consequently, the diagnostic utility of electromyography in this particular case is considered relatively limited.

Imaging studies, including plain radiography, magnetic resonance imaging, and computed tomography (CT), have limited utility for the diagnosis of PS. Although magnetic resonance imaging and CT may occasionally reveal an enlarged piriformis muscle, their primary significance lies in differentiating herniated discs from other vertebral conditions, such as fractures, arthritis, or pathological masses.^[11,15]^

Ultrasound has recently been utilized for the diagnosis and treatment of PS because of its advantages, including real-time, dynamic assessment, and cost-effectiveness. A technique involving identification of the piriformis muscle by connecting the midpoint between the PSIS and sacral hiatus with the upper border of the gluteal tuberosity has been employed to aid in the evaluation and differentiation of PS.^[16,17]^ Siahaan et al showed that if the thickness of the piriformis muscle measured by ultrasound was > 0.9950 cm, the sensitivity and specificity of diagnosing PS were 94.8% and 87.9%, respectively.^[17]^ In this case report, the thickness of the piriformis muscle was 1.03 cm, and no anatomical variation in the sciatic nerve traveling between the piriformis muscles was observed on ultrasound. Considering that the symptoms improved dramatically after piriformis injection, it can be interpreted that piriformis muscle fibers can compress the sciatic nerve in environments with excessive strain on the lower extremity muscles, such as in high G-sorties.

The mechanisms and prevalence of G-induced stress in musculoskeletal symptoms are not yet fully understood. One study demonstrated that pilots with high G-sorties tended to have more lower-limb musculoskeletal symptoms than those with low G-sorties and headquarters, although this difference was not statistically significant (*P* = .064).^[18]^ It is presumed that musculoskeletal pain occurs during and after voluntary muscle contraction of anti-G straining maneuvers to overcome high G-force. Also, such static postures, maintained for prolonged periods, can induce hypertonicity in the gluteal and piriformis muscles, potentially leading to the entrapment of the sciatic nerve.^[19,20]^

Conservative treatment, medication, and physical therapy can be used to treat PS. If there is no response to these treatments, invasive options including injections or surgery may be considered.^[1,8]^ In this case, although the patient was already taking pregabalin and NSAIDs for lumbar radiculopathy, the pain had not been controlled for several days. Therefore, invasive treatment was prioritized.

Ultrasound-guided piriformis injection is a minimally invasive procedure that involves the injection of medication directly into the piriformis muscle using ultrasound guidance. Ultrasound-guided injections are superior to other treatments for PS owing to their precision, safety, and efficacy. Unlike physical therapy, they provide immediate relief, and, compared to oral medications, they minimize systemic side effects through localized drug delivery. They are less invasive than surgery with fewer risks and faster recovery, making them an optimal first-line option for severe or refractory cases.^[21–23]^ This approach allows physicians to obtain images to rule out other diseases and ensure precise delivery of the regimen to the piriformis muscle, thus making it a highly targeted treatment method. Botox, steroids, and analgesic agents such as lidocaine or procaine are commonly used in injection regimens.^[24]^ Several reports have suggested that there is no significant difference in effectiveness based on the type of regimen.^[24,25]^ In this case, lidocaine, which has fewer side effects and is more cost-effective, was used to treat the PS.

## 4. Conclusion

PS can be a debilitating condition for military pilots, impeding their flying abilities and sortie performance. PS should be considered in the differential diagnosis for recurrent neuropathic pain, particularly in high-risk patients such as military fighter pilots with a prior history of lumbar radiculopathy. Ultrasound-guided piriformis injection is an effective and targeted treatment, aiding in ruling out other diseases, alleviating PS symptoms, and enabling pilots to resume their sorties without impediments. Moreover, prospective cohort research with a larger sample of military pilots is necessary to determine the prevalence and incidence of PS.

## Acknowledgments

The authors would like to thank the patient for agreeing to publication of this report.

## Author contributions

**Conceptualization:** Do Yun Kwon, Dong Hyuck Kim.

**Data curation:** Do Yun Kwon, Dong Hyuck Kim.

**Supervision:** Dong Hyuck Kim.

**Writing – original draft:** Do Yun Kwon.

**Writing – review & editing:** Do Yun Kwon, Dong Hyuck Kim.
